# Genomic Studies Reveal Substantial Dominant Effects and Improved Genomic Predictions in an Open-Pollinated Breeding Population of *Eucalyptus pellita*

**DOI:** 10.1534/g3.120.401601

**Published:** 2020-08-11

**Authors:** Saravanan Thavamanikumar, Roger J. Arnold, Jianzhong Luo, Bala R. Thumma

**Affiliations:** * Gondwana Genomics Pty Ltd, Gould building, 116 Daley Road, Acton, ACT 2601, Australia; † China Eucalypt Research Centre, Chinese Academy of Forestry, 30 Renmin Dadao, Zhanjiang, Guangdong 524022, China

**Keywords:** genomic selection, nonadditive effects, prediction accuracy, single-step GBLUP, ABLUP, GenPred, Genomic Prediction, Shared data resources

## Abstract

Most of the genomic studies in plants and animals have used additive models for studying genetic parameters and prediction accuracies. In this study, we used genomic models with additive and nonadditive effects to analyze the genetic architecture of growth and wood traits in an open-pollinated (OP) population of *Eucalyptus pellita*. We used two progeny trials consisting of 5742 trees from 244 OP families to estimate genetic parameters and to test genomic prediction accuracies of three growth traits (diameter at breast height - DBH, total height - Ht and tree volume - Vol) and kraft pulp yield (KPY). From 5742 trees, 468 trees from 28 families were genotyped with 2023 pre-selected markers from candidate genes. We used the pedigree-based additive best linear unbiased prediction (ABLUP) model and two marker-based models (single-step genomic BLUP – ssGBLUP and genomic BLUP – GBLUP) to estimate the genetic parameters and compare the prediction accuracies. Analyses with the two genomic models revealed large dominant effects influencing the growth traits but not KPY. Theoretical breeding value accuracies were higher with the dominance effect in ssGBLUP model for the three growth traits. Accuracies of cross-validation with random folding in the genotyped trees have ranged from 0.60 to 0.82 in different models. Accuracies of ABLUP were lower than the genomic models. Accuracies ranging from 0.50 to 0.76 were observed for within family cross-validation predictions with low relationships between training and validation populations indicating part of the functional variation is captured by the markers through short-range linkage disequilibrium (LD). Within-family phenotype predictive abilities and prediction accuracies of genetic values with dominance effects are higher than the additive models for growth traits indicating the importance of dominance effects in predicting phenotypes and genetic values. This study demonstrates the importance of genomic approaches in OP families to study nonadditive effects. To capture the LD between markers and the quantitative trait loci (QTL) it may be important to use informative markers from candidate genes.

*Eucalyptus pellita* F. Muell, or red mahogany, is a medium-size to tall tree with good form that can grow up to 40 m or more in height and 1 m in diameter (Harwood 1998). Its ecological niche is between rainforest and savannah woodlands in humid and sub-humid tropical environments extending from coastal northern Queensland into southern New Guinea (Vercoe and McDonald 1991; Harwood 1998). Attributes that make *E. pellita* an attractive species for plantations include fast growth, good coppicing ability, adaptability to a range of tropical environments, good resistance to pests and diseases and timber suitable for a variety of products including pulp and paper, veneers and sawn-timber. On account of the species superior resistance to fungal pathogens and reasonably rapid growth in lowland tropical environments, *E. pellita* has found favor as a plantation species for pulpwood production in high rainfall lowland areas on mineral soils (Booth *et al.* 2017). While it was being grown in plantations on a relatively small scale in northern Australia, South East Asia and Brazil up till about 2010, recent burgeoning disease issues with tropical acacia plantation species has led to a surge of commercial planting of the species for short rotation fiber production in moist lowland tropical environments (Nambiar *et al.* 2018). In Indonesia and Malaysia losses of *Acacia mangium* plantation trees, often on catastrophic scales, over recent years due to outbreaks of *Ganoderma* spp. (root rot) (Eyles *et al.* 2008) and *Ceratocystis* spp. (stem canker) fungal pathogens (Tarigan *et al.* 2011) have led to the replacement of this species on a massive scale by *E. pellita* (Mendham *et al.* 2015). In the last 10 years, over 600,000 ha of *A. mangium* plantations in just these two countries alone, have been replaced with those of *E. pellita*, or hybrids of this species (Hardiyanto *et al.* 2018).

Genetic studies using progeny trials of *E. pellita* in SE Asia have revealed moderate heritability for growth and wood traits including pulp yield (Brawner *et al.* 2010; Hung *et al.* 2015). These studies also revealed little genotype by environment interaction (GXE) for these traits. There are however few studies using genomics in *E. pellita*. Recently Müller *et al.* (2017) used SNP markers genotyped with EUChip60K to study genomic selection and genome-wide association studies in *Eucalyptus benthamii* and *E. pellita*. In a previous project (S. Southerton, S. Thavamanikumar, B. Thumma, unpublished), we developed custom marker panels for routine genotyping in several species of *Eucalyptus* including *E. pellita*. These marker panels were developed from candidate genes of growth and wood traits selected from our previous RNA-seq studies (Thumma *et al.* 2012; Thavamanikumar *et al.* 2014) and surveying the literature. For identifying markers, we sequenced samples from extremes of trait distribution that were derived from several populations. Markers with the potential association to the traits were identified by comparing allele frequencies of samples from trait extremes. Markers exhibiting large and consistent allele frequency differences between the trait extremes of different populations were selected to develop the custom marker panels. The marker panels consist of probes to capture short genomic regions spanning the candidate markers. Targeted genotyping by sequencing (TGS) was used to genotype the captured genomic regions. In this study, we used the custom marker panel developed for *E. pellita* to conduct genomic studies.

Several studies in forest trees have tested the efficacy of genomic selection in tree breeding with moderate to high prediction accuracies observed for different traits (Beaulieu *et al.* 2014; Muñoz *et al.* 2014; Bartholomé *et al.* 2016; Thavamanikumar *et al.* 2018; Suontama *et al.* 2019). Application of markers in open-pollinated (OP) families is particularly useful as markers can uncover half-sib and full-sib relationships within the OP families (El-Kassaby *et al.* 2011). This will have a significant impact on the precision and accuracy of the estimated genetic parameters as demonstrated by a number of studies (Bush and Thumma 2013; Gamal El-Dien *et al.* 2016, 2018; Müller *et al.* 2017; Klápště *et al.* 2017; Klápšte *et al.* 2018). In traditional methods, all individuals of an OP family are treated as half-sibs. Therefore, the precision and accuracy of genetic parameters estimated in OP families are in general lower than those estimated in controlled-pollinated (CP) families. This is mainly due to the unrealistic assumption that all individuals of an OP family are half-sibs. In OP populations, the genetic variances and breeding values estimated with best linear unbiased prediction (BLUP) methods using pedigree-based additive or numerator relationships are generally inflated due to overestimation of variance components (Gamal El-Dien *et al.* 2016).

Application of Genomic BLUP (GBLUP) methods using genomic relationship matrices can provide high accuracies of the estimated genetic parameters. This is mainly because markers capture the Mendelian segregation term *i.e.*, covariance among sibs within the families (VanRaden 2008; Zapata-Valenzuela *et al.* 2013) along with the LD between markers and QTL (Habier *et al.* 2010, 2013). The Mendelian sampling/segregation term represents the variation in progeny due to random sampling of alleles of a locus from each parent during meiosis (Avendaño *et al.* 2005). OP families used in tree breeding are in general shallow with minimal connectedness between the families. Markers capture the known contemporary relationships as well as unknown historic relationships and thus can improve the accuracy of genetic parameters estimated with genomic approaches (Powell *et al.* 2010).

However, the main advantage of using markers in OP families is to study the nonadditive effects, something that is not possible with traditional methods. Dominance plays a significant role in the genetic control of growth traits compared to wood traits. Studying dominance and epistatic effects usually requires clonally replicated full-sib families (Costa E Silva *et al.* 2004). Muñoz *et al.* (2014) have demonstrated estimating dominance and epistatic effects accurately using clonally replicated full-sib families. Now however, these effects can be studied in OP families using genomic approaches without the need for special populations. Analysis of nonadditive and epistatic effects in OP families provides huge opportunities for genetic improvement as the production of OP families is simpler and less expensive than the production of clonally replicated CP families. In a first study of this kind, Gamal El-Dien *et al.* (2016) used genomic approaches to study nonadditive effects in an OP population of white spruce. Using GBLUP, they identified significant epistatic genetic variance which was confounded with additive variance in pedigree-based BLUP analysis leading to overestimation of heritability.

In forest trees, several studies have used the GBLUP method to test the accuracy of genomic selection (Zapata-Valenzuela *et al.* 2013; Isik *et al.* 2016; Kainer *et al.* 2018; Chen *et al.* 2019). In the GBLUP, the pedigree-based additive relationship matrix is replaced with a realized genomic relationship matrix (GRM) from markers. As GRM captures both known and unknown (hidden) relationships, accuracies of the GBLUP are generally higher especially in breeding populations with shallow pedigree and disconnected families. However, the GBLUP method can only be applied with the genotyped samples. A typical breeding program contains data on several thousands of individuals. As genotyping every individual is expensive and time-consuming, information from genotyped and non-genotyped samples can be combined using a single-step GBLUP (ssGBLUP) to estimate breeding values (Legarra *et al.* 2009; Misztal *et al.* 2009; Christensen and Lund 2010). In the ssGBLUP, a pedigree derived additive relationship matrix (*A* matrix) is combined with a marker derived genomic relationship matrix (*G* matrix) to generate a combined relationship matrix (*H* matrix). The combined *H* matrix is then used in BLUP analysis to estimate the genetic parameters and breeding values. Thus, in the ssGBLUP, information from both genotyped as well as non-genotyped individuals is used which leads to higher precision and accuracy of the genetic parameters estimated. The genomic relationships of the *G* matrix are transferred to non-genotyped samples through the *A* matrix leading to high accuracies of genetic parameters. Thus, the advantage of ssGBLUP is that all the available information is used optimally (Legarra *et al.* 2009).

While, there are many studies using the ssGBLUP in animal breeding there are only a few such studies in forest trees (Ratcliffe *et al.* 2017; Cappa *et al.* 2017, 2019; Klápšte *et al.* 2018). In one of the first studies to implement this method in tree breeding, Cappa *et al.* (2017) found increased accuracies of breeding values with the ssGBLUP compared to pedigree-based ABLUP method. Most of the genomic studies in plants and animals have used ssGBLUP with additive effects to analyze genetic parameters and to test accuracies of genomic predictions. With the availability of methods to calculate genomic dominance relationship matrices it is straight-forward to include dominance effects in the ssGBLUP models. However, the main problem for using dominance effects in single-step analyses is that it is computationally not feasible to invert genomic dominance relationship matrices from large numbers of samples (Ertl *et al.* 2018). The convergence of single-step methods is one of the main problems encountered when a large number of samples are used. Inclusion of dominance deviations in the model in addition to breeding values will exacerbate the problem of convergence in real-world systems (Ertl *et al.* 2018). We tested the ssGBLUP model with dominance effects in this study as the number of genotyped trees are small compared to the total number of trees used and as no pedigree-based dominance matrix needs to be generated from OP families making the inversion of the combined matrix relatively easy.

In this study, we used 244 OP families of *E. pellita* from two trials (at Dongmen and Yulin in southern China) to estimate genetic parameters with genomic approaches. We used ssGBLUP and GBLUP to study additive and nonadditive genetic variances influencing growth traits and pulp yield. The main objectives of this study were: 1) to estimate genetic parameters using ssGBLUP and GBLUP methods with additive and dominance effects; and, 2) to compare the prediction accuracies from different models.

## Materials and Methods

### Trial sites

The *E. pellita* trials used in this study were both located in southern Guangxi Province, China. One was at Dongmen Forest Farm at approximate latitude 22°23’ N, longitude 107°52’ E and an elevation of 120 m above sea level. The other trial was planted on land belonging to Yulin Forestry Research Institute at around latitude of 22°39′N, longitude 110°09′ E and altitude of 400 m above sea level. The Dongmen trial comprised 6 complete replicates with each family represented by a 4-tree-row plot within each replicate and with randomization of the layout of family plots within each replicate. The Yulin trial was laid out with 30 complete replicates of single tree plots, also with randomization of the layout of family plots within each replicate. At both sites, the spacing was 2.0 m (between trees) × 3.5 m (between rows).

The trial at Yulin included 244 open-pollinated families from plus-trees selected for growth, stem form and typhoon tolerance in three 1^st^ generation *E. pellita* provenance family trials established during the 1990s to early 2000s in southern China. The trial at Dongmen included 229 OP families that were a subset of those included in the Yulin trial. Details of these seedlots (OP families) are provided in Table 1. Fewer families were included in Dongmen trial due to a limited availability of seed of some families combined with imperfect germination of some family seedlots; this led to inadequate numbers of seedlings to enable both trials to be established with the full complement of families.

**Table 1:** Details of the *E. pellita* seedlots (OP families) established in the second-generation family trials at two sites in Guangxi, China

Seed source (field trial) details				No. of selections represented in 2^nd^ generation trials	
				(as open-pollinated families)	
Trial location		Year established	No of provenances/families	Yulin	Dongmen
1	Leizhou provenance-family trial	1998	14/244	156	148
2	Dongmen provenance-family trial number E138	2003	7/118	48	46
3	Dongmen provenance-family trial number E53	1996	9/80	40	35
**Total**				**244**	**229**

The trial at Dongmen was planted in July 2011 and that at Yulin was planted in June 2011. Both trials were assessed for survival and growth at age of approximately 41/2 years (around 54 months); all surviving trees were measured for total height (Ht) and for diameter over bark at a height of 1.3 m (Diameter at Breast Height - DBH).

The measurements of diameter and total height were used to calculate conical tree volumes (over bark) for all trees using the following formula:

$$VOL=\pi \times {\left(DBH/2\right)}^{2}\times \frac{Ht}{3}$$

Where VOL = index of over bark tree volume in dm^3^, π = the mathematical constant Pi = 3.14, DBH = diameter at breast height (1.3 m) over bark in dm, Ht = total tree height in dm.

At approximately age 5 years (around 61 months), wood and DNA samples were taken from a subset of trees in each trial that included 28 families in common between the trials, ∼8 trees sampled per family from each site. This provided 232 and 236 trees for sampling from Dongmen and Yulin respectively, making a total of 468 trees sampled across the two sites. The reason for just 28 families being sampled was a combination of: i) resources available for this study, primarily costs of DNA genotyping and KPY determinations; and, ii) the number of families available that had ≥8 trees surviving (and not stunted or wind damaged) at each site (both trials had incurred considerable wind/typhoon damage prior to age 5 years). None of the 28 parents of the families that were genotyped, were themselves sampled for genotyping or KPY determinations.

The DNA samples were obtained as stem cambium scrapings. These were obtained by removing a window of bark (size of around 3 × 3 cm) at 1.3 m height on the stem facing the row direction, then scrapping cambial tissue using a sharp wood chisel into a labeled 2 ml centrifuge tube containing standard CTAB buffer. The wood samples were taken in the form of drill fras. This was obtained using an 11 mm drill bit that was drilled into the tree stem to a depth of approximately 6 cm in the place where cambial scrapping had been taken and hence was free of bark. The drill fras samples were air-dried and then shipped to Forest Quality Pty Ltd in Tasmania, Australia, for oven drying, grinding and then the prediction of kraft pulp-yield (KPY) individually for each sample using Near Infra-Red spectral analyses. DNA from cambial scrapes were extracted and genotyped at Gondwana Genomics Pty Ltd, Canberra, using *E. pellita* marker panel. The *E. pellita* marker panel consisted of single nucleotide polymorphism (SNP) markers and small biallelic insertion deletions (INDEL) markers from 2,000 candidate genes. The markers included in the marker panel were distributed across all the 11 chromosomes. Markers were preselected for potential association with various traits. Preselection of the SNPs was based on allele frequency differences from sequencing pooled samples representing the core germplasm of *E. pellita* breeding used in SE Asia. After applying different filters (*e.g.*, minor allele frequency (MAF) > 5%, SNP call rate of > 90%), in total 2,023 markers were genotyped in 423 trees which were then used in all subsequent genomic analyses (Table S2).

### Statistical models

Prior to analyses, the phenotypic trait data for DBH, total height, tree volume (over bark) and KPY were adjusted to account for site differences. For this, data from each trait from Yulin were adjusted using PROC STANDARD procedure of SAS software to have the same mean and standard deviation as the same trait at Dongmen. Phenotypic data were adjusted for trial design effects by considering family by rep as a random effect. Adjusted phenotypes were used in all subsequent analyses.

Three models, traditional ABLUP, and two genomic models ssGBLUP and GBLUP were used to estimate the breeding values. For ssGBLUP models, trait data of all trees was used, for GBLUP trait data of only genotyped trees was used. For ABLUP trait data of all trees as well as just the genotyped trees was used in two different analyses.

### ABLUP

y=Xμ+Za+ϵ

Where y is the phenotype adjusted for site effects, μ is the intercept, a is a vector of the random additive genetic effects of individual trees, ϵ is the vector of random residual effects. X and Z are the incident matrices relating to fixed and random effects, a is distributed as $a∼N(0,A\sigma^{2}a)$ where $\sigma^{2}a$ is the additive genetic variance and A is the average additive genetic relationship matrix from pedigree, ϵ is distributed as $\epsilon ∼N(0,I\sigma^{2}\epsilon )$ where I is an identity matrix and $\sigma^{2}\varepsilon$ is the residual variance.

### GBLUP

y=Xμ+Zg+ϵ

The GBLUP model is the same as the ABLUP except that the A matrix is replaced by G matrix derived from the markers to estimate molecular breeding values (MBVs). In GBLUP the g vector is distributed as $g∼N(0,G\sigma^{2}g)$ where $\sigma^{2}g$ is the additive genetic variance and G is the marker derived additive genomic relationship matrix.

### GBLUP-AD

$$y=X\mu +Z_{1}g+Z_{2}d+\epsilon$$

Where d is the vector of dominance genetic effects distributed as $d∼N(0,D\sigma^{2}d)$, $\sigma^{2}d$ is the dominance genetic variance and D is the marker derived dominance genomic relationship matrix.

The molecular genetic values (MGV) are then estimated as MGV=g+d

### ssGBLUP

In ssGBLUP, similar to the GBLUP, the *A* matrix is replaced by *H* matrix from combing pedigree and genotype information to estimate genomic estimated breeding values (GEBVs).

### Genomic relationship matrices

#### Additive relationship matrix (G):

Additive genomic relationship matrix is based on the VanRaden method (VanRaden 2008) and derived as follows:

G=WaW’a2∑​pj=1mj(1−pj)

Where W is the incidence matrix of the SNP markers with $W_{aij}=\{2-2p_{j},1-2p_{j},-2p_{j}\}$, where $W_{aij}$ represents the elements of $W_{a}$ matrix at i^th^ row and j^th^ column. $p_{j}$ is the allele frequency of j^th^ marker.

#### Dominance genomic relationship matrix (D):

Dominance genomic relationships matrix is based on (Vitezica *et al.* 2013; Aliloo *et al.* 2017; Zhang *et al.* 2019) and derived as follows:

D=WdW’d4∑​(pj(1−pj))j=1m2

$W_{d}$ is expressed as

$$W_{dij}=\{-2{\left(1-p_{j}\right)}^{2},2p_{j}(1-p_{j}),-2p_{j}^{2}\}$$

Where $W_{dij}$ represents the elements of $W_{d}$ matrix at i^th^ row and j^th^ column.

#### Combined matrix (H matrix) – additive:

We used HIBLUP (Yin *et al.* 2019) package of R software to develop the H matrix. The combined H-matrix for ssGBLUP was developed using the following equation.

$$H=\left(\begin{array}{cc} A_{11}-A_{12}A_{22}^{-1}A_{21}+A_{12}A_{22}^{-1}GA_{22}^{-1}A_{21} & A_{12}A_{22}^{-1}G\\ GA_{22}^{-1}A_{21} & G+\alpha A_{22} \end{array}\right)$$

For this, individuals were assigned to different groups based on available information; the group with the subscript “1” represent individuals that only had pedigree information and the group with the subscript “2” represent individuals that had both pedigree and genomic information. A11 and A22 represent relationships among individuals within the group “1” and the group “2” respectively, A12 represents relationships among individuals between the group “1” and “2”, and A21 is the transpose of A12.

To have the same scale between G and A22 the following adjustment was made to the *G* matrix Ga=βG+αThe adjustment factors β and α were derived from the following equation (Christensen *et al.* 2012):

Avg.diag(G) β+α=Avg.diag(A22) andAvg.offdiag(G) β+α=Avg.offdiag(A22)

Where: Avg.diag is the average of diagonals and Avg.offdiag is the average of off-diagonal elements.

#### Combined matrix (HD matrix) – dominance:

The combined HD matrix with dominance (Ertl *et al.* 2018) was developed using the same equation as the additive model except that the additive relationship matrices were replaced with dominance matrices as follows:

$$HD=\left(\begin{array}{cc} AD_{11}-A_{D12}AD_{22}^{-1}A_{D21}+AD_{12}AD_{22}^{-1}G_{D}A_{D22}^{-1}A_{D21} & A_{D12}A_{D22}^{-1}G_{D}\\ G_{D}A_{D22}^{-1}A_{D21} & G_{D}+\alpha A_{D}{}_{22} \end{array}\right)$$

All the above models are fitted in R (www.r-project.org) with HIBLUP (https://hiblup.github.io/) (Zhang *et al*. 2019), sommer (Covarrubias-Pazaran 2016) and breedR (Munoz and Sanchez 2014) packages. Sommer and breedR packages were used for GBLUP analyses. HIBLUP and breedR were used for ssGBLUP analyses. H, HD matrix, additive and dominance relationship matrices from HIBLUP were used in sommer to estimate genetic variances and heritabilities.

Narrow-sense heritability (h^2^) was estimated as $h^{2}=\sigma_{g\;}^{2}/(\sigma_{g}^{2}+\sigma_{d}^{2}+\sigma_{\varepsilon }^{2})$. Where $\sigma_{g\;}^{2}$is additive genetic variance; $\sigma_{d}^{2}$ dominance variance; and, $\sigma_{\varepsilon }^{2}$ residual. Dominance to total variance ratio (d^2^) was estimated as $d^{2}=\sigma_{d\;}^{2}/(\sigma_{g}^{2}+\sigma_{d}^{2}+\sigma_{\varepsilon }^{2})$. Broad-sense heritability was estimated as $H^{2}=(\sigma_{g\;}^{2}+\sigma_{d}^{2})/(\sigma_{g}^{2}+\sigma_{d}^{2}+\sigma_{\varepsilon }^{2})$.

### Theoretical accuracy of breeding values

Theoretical accuracies of breeding values from ABLUP, ssGBLUP-A and ssGBLUP-AD models using all the trees were estimated with the following expression.

$$r=\sqrt{\frac{1-PEV}{\sigma_{a}^{2}(1+F_{i})}}$$

Where PEV is the prediction error variance from diagonal elements of the matrix from the mixed model equation (Gilmour *et al.* 1995), $F_{i}$ is the inbreeding coefficient of tree *i*.

### Prediction accuracies and predictive abilities from cross-validation

In addition to theoretical breeding value accuracies, we have estimated prediction accuracies with cross-validation. Prediction accuracy is estimated as the Pearson correlation between breeding values/genetic values estimated with all trees and the predicted breeding values/genetic values from cross-validation. The prediction accuracies were estimated for all trees as well as for only the genotyped trees. Similarly, predictive ability (PA) is estimated within the genotyped trees as the Pearson correlation between the adjusted phenotypes and the predicted breeding values/genetic values from cross-validation.

Cross-validation tests were performed to test the accuracies and predictive abilities of different models. Three types of cross-validations were performed; random folding, balanced family folding and family folding. Random folding tests were performed in all samples and in genotyped samples separately. The balanced family folding and the family folding tests were performed only in the genotyped samples. In random folding, trees were randomly split into training and validation populations. A 10-fold cross-validation was performed in random folding. In the balanced family folding, a minimum of 20% of trees from each family were used in the validation population. To have a minimum of 20% trees/family, a fivefold cross-validation was performed in balanced family folding. In family folding, entire families were removed from the training population to remove genetic relatedness between training and validation populations. Thus, for family folding cross-validation a 24-fold test was performed. Even though the genotyped trees are from 28 families, only 24 families were tested in validation as individuals from four families were less than eight. In family folding, the ABLUP model was not tested as predicted breeding values would have been equal to the mean of the training model for each predicted family.

### Data availability

The pedigree and trait data used in this study are in Table S1. The marker genotype data used in this study are in Table S2. Supplemental material available at figshare: https://doi.org/10.25387/g3.12782567.

## Results

### Analyses with all trees (genotyped and non-genotyped)

#### Estimating genetic parameters with ABLUP and ssGBLUP:

Pedigree-based ABLUP and marker and pedigree-based single-step GBLUP (ssGBLUP) were used to estimate genetic parameters of growth traits (DBH, Ht and Vol) for which trait data were available from 5,742 trees. Narrow-sense heritability estimates for all three models (ABLUP, ssGBLUP_A and ssGBLUP_AD) are similar among each of the three traits (Table 2). While the narrow-sense heritability estimates are similar between DBH and Ht, they are however lower for Vol. Analysis of ssGBLUP with dominance (ssGBLUP_AD) revealed significant dominance effects for all three traits resulting in high broad-sense heritability estimates (Table 2). Meanwhile the dominance ratios (d2) ranged between 0.39 and 0.50 among the three traits and there was more than 100% increase in broad-sense heritability estimated with the ssGBLUP_AD model compared to narrow-sense heritability estimated with the ssGBLUP_A model.

**Table 2:** Genetic parameters of three growth traits estimated with different models (numbers given in parentheses represent the parameter standard errors)

	DBH			Ht			Vol		
	ABLUP	ssGBLUP_A	ssGBLUP_AD	ABLUP	ssGBLUP_A	ssGBLUP_AD	ABLUP	ssGBLUP_A	ssGBLUP_AD
h^2^	0.34 (0.04)	0.35 (0.04)	0.33 (0.04)	0.36 (0.04)	0.38 (0.04)	0.38 (0.04)	0.28 (0.04)	0.28 (0.04)	0.26 (0.04)
d^2^	NA	NA	0.50 (0.08)	NA	NA	0.42 (0.08)	NA	NA	0.39 (0.09)
H^2^	NA	NA	0.83 (0.08)	NA	NA	0.80 (0.09)	NA	NA	0.66 (0.09)
LogL	−2744.33	−2739.62	−2729.40	−2754.87	−2752.29	−2746.83	−2787.86	−2783.87	−2771.73
AIC	5490.65	5481.23	5460.79	5511.73	5506.57	5495.65	5577.71	5569.74	5545.46

h^2^, narrow-sense heritability, d^2^, dominance to total variance ratio, H^2^, broad-sense heritability, logL, log-likelihood, AIC, Akaike information criterion.

Inclusion of the dominance effect in the model (ssGBLUP_AD) resulted in the reduction of error variance while maintaining the additive genetic variance similar to that of ABLUP and ssGBLUP_A models. Inclusion of genomic data also improved model fit over pedigree model as indicated by goodness-of-fit statistics (Akaike Information Criterion - AIC). Among the two genomic models (ssGBLUP_A and ssGBLUP_AD) inclusion of dominance in the model further improved the model fit for all three traits (Table 2).

#### Comparison of pedigree and genomic relationship matrices of parents:

**Figure 1:**
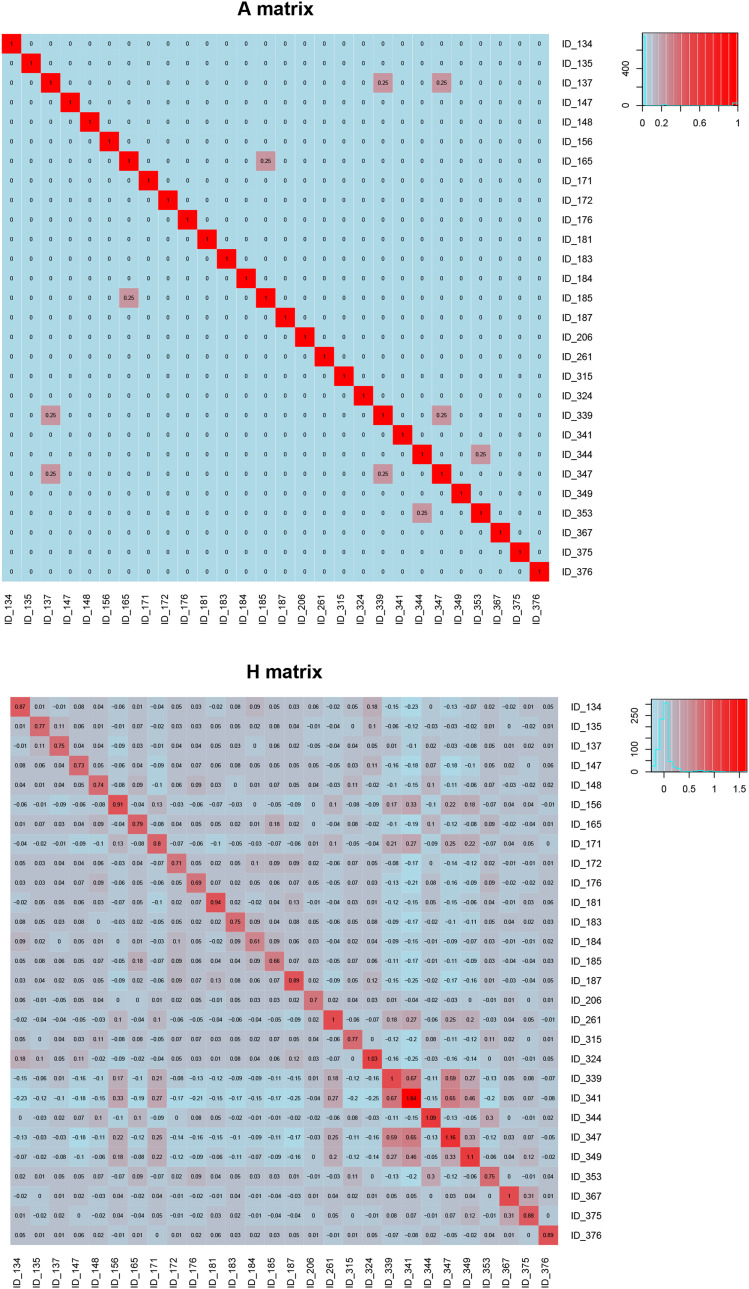
Comparison of pedigree-based *A* matrix and combined *H* matrix of the 28 parents with the genotyped progeny. Heatmaps with the genetic relationships are shown in each matrix. Each matrix represents pair-wise relationships between the 28 parents.

In ssGBLUP, genomic relationships captured by the markers are projected on to non-genotyped individuals. This results in a denser additive relationship matrix among the non-genotyped samples compared to the pedigree-based numerator relationship matrix (NRM). To demonstrate this, we compared the additive relationship matrix from pedigree (*A* matrix) with *H*-matrix used in ssGBLUP for 28 parents which had genotyped progeny; but the parents themselves were not genotyped (Figure 1). This comparison showed that the additive relationship matrix from pedigree was sparse with only a few high-level relationships, while the *H*-matrix was dense with extensive relationships among the 28 parents unraveled by the genotyped progeny even though none of the parents were genotyped. These dense relationships among non-genotyped samples provide the basis for higher accuracies of ssGBLUP compared to the ABLUP.

According to the pedigree file (Table S1), among the 28 families, there are seven parents derived from three families (three grandmothers). Three parents 137, 339 and 347 are from one family, therefore, are half-sibs; 165 and 185 are from a second family and 344 and 353 are from a third family. Except for one parent (137), all other parental relationships are confirmed with *H*-matrix (Figure 1). This matrix revealed that while there are a few high pair-wise relationships among the parents (9% of parents with genomic relationships > 0.10), most of the relationships among the parents are low (92% of parents with genomic relationships < 0.10). One of the parents (341) is identified as an inbred in the *H*-matrix (Figure 1). Examining the GRM of the genotyped progeny indicated high inbreeding among the progeny derived from this parent reflecting the high inbreeding of the parent detected with the *H*-matrix (data not shown). This illustrates that *H*-matrix can be used to detect deep genetic relationships among the parents even when they are not genotyped.

#### Theoretical breeding value accuracies:

Theoretical breeding value accuracies were estimated for ABLUP and ssGBLUP models using all trees. Theoretical breeding value accuracies are generally higher with the two ssGBLUP models (ssGBLUP-additive and ssGBLUP-dominance) compared to pedigree-based ABLUP model for all three growth traits. Among the two ssGBLUP models, breeding values accuracies are generally higher for the model with the dominance effects (Table 3). Accuracies of the genotyped progeny and parents with the genotyped progeny are higher than non-genotyped progeny and all parents. Within the non-genotyped progeny, inclusion of dominance effects in ssGBLUP improved the accuracies compared to ABLUP and ssGBLUP-additive models for all three traits. As expected, breeding value accuracies of parents are higher than those of the progeny.

**Table 3:** Mean theoretical breeding value accuracies among the three growth traits based on ABLUP, ssGBLUP (additive) and ssGBLUP(dominance) in *E. pellita*

	DBH			Ht			Vol		
	ABLUP	ssGBLUP additive	ssGBLUP dominance	ABLUP	ssGBLUP additive	ssGBLUP dominance	ABLUP	ssGBLUP additive	ssGBLUP dominance
All parents	0.81	0.81	NA	0.82	0.83	NA	0.78	0.78	NA
Parents*^a^*	0.84	**0.86**	NA	0.86	**0.87**	NA	0.82	**0.84**	NA
genotyped progeny	0.63	0.68	**0.76**	0.64	0.70	**0.72**	0.61	0.63	**0.69**
non-genotyped progeny	0.64	0.65	**0.72**	0.66	0.67	**0.68**	0.60	0.60	**0.64**
all progeny	0.64	0.65	**0.73**	0.66	0.68	**0.68**	0.60	0.60	**0.64**

a Parents with the genotyped progeny; The highest accuracies among the three models within each trait are highlighted in bold.

#### Prediction accuracies with cross-validation using all trees:

**Figure 2:**
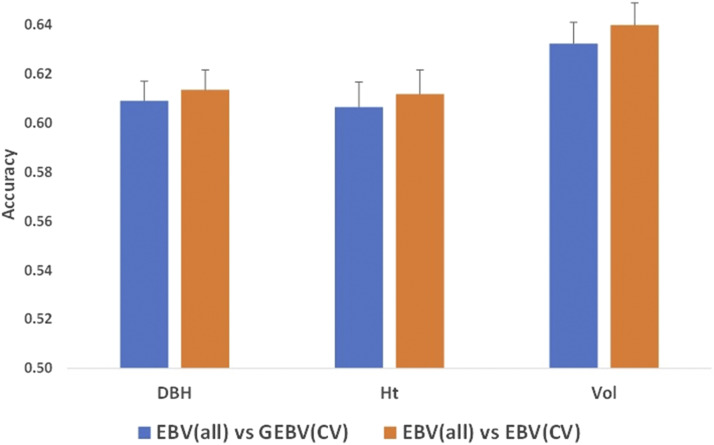
Prediction accuracies of growth traits from cross-validation with random folding using all trees. EBV - Estimated breeding value, GEBV - genomic estimated breeding value

Accuracies of predicted breeding values from ABLUP were also assessed by correlating EBVs estimated using all samples (EBV_all) with EBVs from cross-validation (EBV_CV). Similarly, accuracies of GEBVs from ssGBLUP were assessed by correlating GEBVs estimated using all samples (GEBV_all) with the GEBVs from cross-validation (GEBV_CV). Cross-validation breeding values from the two methods (GEBV_CV and EBV_CV) were correlated with EBVs_all to compare the accuracies of GEBVs and EBVs (Figure 2). Accuracies between the traits were similar for genomic estimated breeding values (GEBVs) estimated with ssGBLUP and estimated breeding values (EBVs) estimated with ABLUP. In contrast to the theoretical accuracies, the accuracies of Vol (0.63) were higher than the other two growth traits (0.61). Similar results were observed by estimating accuracies as a correlation between GEBV (all) and GEBV/EBV (CV).

### Analyses within the genotyped trees

#### Estimating genetic parameters with GBLUP:

As less than 8% of the trees (423 trees from 28 families out of total 5742 trees from 244 families) with trait data used in ssGBLUP were genotyped, estimates of genetic parameters and breeding values were mainly influenced by non-genotyped trees resulting in these estimates being similar between ABLUP and ssGBLUP (Figure 2). To gain a better understanding of the influence of genomic data we performed GBLUP using only the genotyped samples. While the accuracy of genetic parameters estimated with the reduced number of samples may not be accurate, we wanted to compare the genetic parameters between different genomic models and to confirm the significant dominance results of ssGBLUP. Additionally, KPY was not used in ssGBLUP as it was only measured in the genotyped samples.

Similar to the results of ssGBLUP, significant dominance effects were observed for all three growth traits but not for KPY resulting in moderate to high broad-sense heritability (H^2^) for growth traits (Table 4). Also, the fit of the models that included dominant effects (GBLUP-AD) was better compared to the models with additive effects (P_A, GBLUP_A) for all traits except for KPY. Among the three growth traits, estimates of dominance ratios (d^2^) were higher for DBH and Vol compared to Ht and the error associated with d^2^ was higher for Ht. For DBH, h^2^ with ABLUP (P_A) was close to zero. For the other three traits (Ht, Vol and KPY) h^2^ estimates with ABLUP were higher than those of GBLUP.

**Table 4:** Genetic parameters estimated with different models using 423 genotyped trees. (numbers given in parentheses represent the parameter standard errors)

	DBH			Ht			Vol			KPY		
	P_A	GBLUP_A	GBLUP_AD	P_A	GBLUP_A	GBLUP_AD	P_A	GBLUP_A	GBLUP_AD	P_A	GBLUP_A	GBLUP_AD
h^2^	0.01 (0.08)	0.07 (0.05)	0.017 (0.03)	0.49 (0.19)	0.13 (0.06)	0.11 (0.06)	0.14 (0.11)	0.08 (0.06)	0.02 (0.02)	0.43 (0.18)	0.10 (0.06)	0.10 (0.07)
d^2^	NA	NA	0.62 (0.12)	NA	NA	0.32 (0.27)	NA	NA	0.70 (0.09)	NA	NA	0.05 (0.30)
H^2^	NA	NA	0.64 (0.12)	NA	NA	0.43 (0.24)	NA	NA	0.72 (0.09)	NA	NA	0.15 (0.32)
LogL	−202.48	−199.45	−184.22	−194.06	−196.13	−191.18	−201.98	−198.59	−181.56	−195.52	−201.27	−201.27
AIC	406.96	400.90	370.44	390.12	394.26	384.37	405.96	399.19	365.13	393.05	404.54	404.53

h2, narrow-sense heritability, d2, dominance to total variance ratio, H2, broad-sense heritability, logL, log-likelihood, AIC, Akaike information criterion.

#### Predictive abilities and accuracies from cross-validation using the genotyped trees:

We next compared the prediction accuracies within the genotyped samples to assess the performance of the different models. Predictive abilities were estimated by correlating breeding values/genetic values from cross-validation with the adjusted phenotypes. Prediction accuracies were assessed by correlating breeding values/genetic values from cross-validation with breeding/genetic values estimated with all the genotyped samples. Genomic breeding values estimated with GBLUP are indicated as molecular breeding values (MBVs) and the total genetic values (additive and non-additive effects) estimated with GBLUP are indicated as molecular genetic values (MGVs). Three types of cross-validation tests, random folding, balanced family folding and family folding (in which the entire family was left out from training samples) are used for testing predictive abilities and prediction accuracies.

Cross-validation results from random folding and balanced family folding are similar between each other compared to those from family folding. Predictive abilities of MGVs with random folding and balanced family folding (correlation between adjusted phenotype and MGVs from cross-validation) are higher for growth traits than the MBVs and EBVs (Table 5). For KPY however, the predictive ability is higher for EBVs. Prediction accuracies with random folding and balanced family folding are higher for the genomic models compared to ABLUP for all traits. Among the two genomic models, accuracies are higher for MBVs than MGVs.

**Table 5:** Predictive abilities (PA) and prediction accuracies of different models using 423 genotyped samples. (numbers given in parentheses represent the parameter standard errors)

Folding	*^a^*DBH-MGV	DBH-MBV	Vol-MGV	Vol-MBV	Vol-EBV	Ht-MGV	Ht-MBV	Ht-EBV	KPY-MGV	KPY-MBV	KPY-EBV
CV – random											
PA	0.33(0.04)	0.13(0.03)	0.33(0.04)	0.16(0.03)	0.08(0.02)	0.28(0.04)	0.19(0.05)	0.24(0.05)	0.13(0.05)	0.21(0.08)	0.25(0.04)
Accuracy	0.77(0.02)	0.81(0.01)	0.70(0.03)	0.78(0.02)	0.50(0.04)	0.71(0.02)	0.83(0.01)	0.57(0.03)	0.70(0.03)	0.77(0.03)	0.54(0.03)
CV – balanced family											
PA	0.28(0.03)	0.11(0.07)	0.29(0.04)	0.14(0.07)	0.08(0.04)	0.27(0.04)	0.20(0.05)	0.25(0.03)	0.14(0.02)	0.14(0.02)	0.24(0.01)
Accuracy	0.73(0.03)	0.77(0.02)	0.68(0.03)	0.81(0.02)	0.56(0.02)	0.67(0.03)	0.77(0.01)	0.52(0.03)	0.68(0.02)	0.70(0.02)	0.53(0.02)
CV - family											
PA	0.34(0.04)	0.07(0.06)	0.31(0.03)	0.01(0.06)	NA	0.12(0.05)	0(0.05)	NA	0(0.05)	0.03(0.06)	NA
Accuracy	0.76(0.03)	0.66(0.04)	0.70(0.03)	0.63(0.04)	NA	0.56(0.04)	0.49(0.06)	NA	0.54(0.05)	0.59(0.04)	NA

a DBH-EBV could not be tested due to low additive variance. MGV: Molecular genetic values, MBV: Molecular breeding values, EBV: estimated breeding values.

Predictive abilities of MBVs (correlation between adjusted phenotype and MBVs from cross-validation) were zero or close to zero for all traits with family folding (Table 5). Predictive abilities of MGVs are however higher for growth traits. Among the growth traits, DBH and Vol had higher predictive abilities compared to Ht. Predictive abilities of DBH and Vol are similar between the three cross-validation tests (random folding, balanced family folding and family folding). In contrast to the random folding and balanced family folding, predictive accuracies are higher with the MGVs than with the MBVs for all traits except for KPY in family folding. There is a substantial decrease in the MBV accuracies of all traits and in the MGV accuracies of Ht and KPY in family folding compared to random folding and balanced family folding. However, MGV accuracies between random folding and family folding are similar for DBH and Vol. Among all the traits, DBH and Vol had higher accuracies compared to the other two traits in cross-validation with family folding.

## Discussion

### Estimating dominance with ssGBLUP and GBLUP models

In this study, we used single-step GBLUP (ssGBLUP), GBLUP and ABLUP models to estimate genetic parameters and to test the prediction accuracies of different traits in an *E. pellita* breeding population. Most of the previous studies in forest trees which used ssGBLUP have included only the additive effects in the model (Ratcliffe *et al.* 2017; Cappa *et al.* 2017, 2019; Klápšte *et al.* 2018). In contrast, we used additive and dominance effects in ssGBLUP model to study the genetic parameters and to test prediction accuracies of different traits. This study, therefore, represents one of the first empirical studies to include dominance in ssGBLUP. Inclusion of dominance in the model revealed large effects of dominance for the three growth traits. These large dominance effects are further confirmed with the GBLUP analysis performed within the genotyped samples. This demonstrates that one of the advantages of applying markers in OP families is to identify non-additive effects, which is not possible with traditional pedigree-based BLUP models. The substantial dominance effects observed for growth traits may be due to a large number of families (244) with an average of 37 trees per family used in this study. This is also reflected in the low standard errors of dominance ratios estimated for the three growth traits in ssGBLUP-AD (Table 2) and for DBH and Vol in GBLUP-AD model (Table 4). The large dominance effects could also be due to hidden full-sibs within the OP families. Close examination of the GRM of the genotyped progeny samples revealed several full-sibs within the OP families (data not shown).

In ssGBLUP, genomic relationships captured by the markers are projected on to non-genotyped samples leading to the high accuracy of the breeding values estimated. Inclusion of dominance effects in the model improved the theoretical breeding value accuracies of all three growth traits compared to the models with only the additive effects. Theoretical breeding value accuracies estimated using parameters from mixed-model equations (MME) reflect the stability of predictions and the amount by which the breeding values will change when more information is available (Bijma 2012). In addition to theoretical breeding value accuracies, prediction accuracies were also tested by correlating breeding values estimated using all trees with those from cross-validation. High accuracies were observed with cross-validation (Figure 2). However, the accuracies among different models are similar in cross-validation. As dominant effects cannot be estimated with the pedigree relationships of OP families, genomic dominance relationships captured by the markers are used for estimating dominance effects of non-genotyped samples of *H*-matrix. The dominance effects of ssGBLUP in this study are therefore mainly derived from the samples that have been genotyped which are transmitted to non-genotyped samples through pedigree relationships. However, the number of genotyped trees are a small fraction (< 10%) of the total number of trees used in this study. This could be one of the reasons for observing similar accuracies between ssGBLUP and pedigree-based ABLUP models with cross-validation (Figure 2). Increasing the proportion of genotyped samples may improve the precision of genetic parameters and prediction accuracies of ssGBLUP as demonstrated by Ratcliffe *et al.* (2017) in *Picea glauca*.

### Genetic parameters and heritability of traits

To better understand the effects of dominance in genomic models, we performed genomic analyses using only the genotyped trees. Genetic parameters and heritability estimated in the genotyped trees are in general lower than those using all trees reflecting the sampling bias due to small sample size and the small number of families in the genotyped trees. However, high heritability estimates were observed for Ht (0.49 *vs.* 0.13, Table 4) and KPY (0.43 *vs.* 0.10, Table 4) with ABLUP compared to GBLUP. This may reflect the overestimation of additive variance with ABLUP as reported in several studies (Muñoz *et al.* 2014; Gamal El-Dien *et al.* 2016). The substantial dominance effects of growth traits observed with ssGBLUP using all trees are confirmed with the GBLUP analysis using only the genotyped trees. GBLUP analysis, however, revealed that the dominance effects are not significant for KPY indicating differences in the genetic architecture of growth and wood chemistry traits. Several other studies have also indicated that the dominance effects are not significant for wood traits (Gamal El-Dien *et al.* 2018; Tan *et al.* 2018; Chen *et al.* 2019). The genomic heritability of Ht from ssGBLUP (0.38, Table 2) in this study is higher than that observed for this trait (0.26) in *E. pellita* by Müller *et al.* (2017). However, in their study, they observed higher genomic heritability for DBH (0.47) compared to that observed for this trait in the current study (0.35, Table 2).

Estimates of variance components and heritability indicate that in ssGBLUP_AD model, the dominance variance is extracted from error variance leaving the magnitude of additive variance similar to that of additive models (Table 2). These results are also reflected to some extent in GBLUP-AD model using the genotyped trees (Table 4). Similar results were reported by Gamal El-Dien *et al.* (2018) in an OP population of interior spruce indicating that the dominant effects are not confounded by additive effects. However, in their study additive and dominance variances estimated with GBLUP_A and GBLUP_AD models were similar resulting in similar prediction accuracies between the two models. In the current study, however, there is 12 to 50% increase in dominance variance for growth traits with ssGBLUP-AD compared to additive variance (Table 2) resulting in a substantial increase in the predictive ability and genetic value accuracy of GBLUP_AD model compared to GBLUP_A model in family folding.

### Predictions with dominance

Predictive abilities with GBLUP_AD model are higher (>100% over additive models) for DBH and Vol with both random folding and family folding methods of cross-validation (Table 5). This is expected as the phenotype includes additive, non-additive and error variances. Similarly, the predictive accuracies of genetic values are higher with GBLUP_AD model for growth traits in family folding cross-validation (Table 5). These results are in contrast to the findings from several studies in forest trees. In several studies with CP and OP families, predictive abilities and accuracies with GBLUP_AD model have either improved marginally or remained similar to additive models despite the detection of significant dominance effects (Bouvet *et al.* 2016; Gamal El-Dien *et al.* 2018; Resende *et al.* 2017; Tan *et al.* 2018). In all these studies, the estimated dominance effects are either similar to additive effects or lower than the additive effects resulting in minimal or no improvement in prediction accuracy with GBLUP_AD models. Using simulation studies, de Almeido Filho *et al.* (2016) have observed that additive-dominance prediction models will be better than additive models, only when high dominance ratios are detected. In this study, the high dominance ratios observed for the three growth traits (Tables 2 and 4) may explain the high predictive abilities and prediction accuracies of GBLUP_AD models compared to that of the additive models. Similar results were also observed by Chen *et al.* (2018) for tree height in Norway Spruce.

### Cross-validation with high and low relationships between training and validation populations

Results of cross-validation with random folding, balanced family folding and family folding revealed different patterns. Predictive abilities and prediction accuracies are higher in random folding and balanced family folding than in family folding (Table 5). However predictive abilities and genetic value accuracies (GBLUP_AD) are similar between the three methods for DBH and Vol. The high dominance effects observed for these two traits in this study may explain the stability of predictive ability and genetic value accuracy between the three cross-validation methods. These results are in contrast to those observed by Müller *et al.* (2017). They observed that the predictive ability of DBH in *E. pellita* was reduced by more than half (from 0.35 to 0.15) when the relationships between training and validation samples are minimized. However, in their study Müller *et al.* (2017) did not study dominance in *E. pellita*.

One of the main reasons for the differences in accuracies between the three methods of cross-validation may be due to the relationships between training and validation samples used. Accuracy of genomic selection is influenced by genomic relationships as well as marker-trait associations captured by the markers (Habier *et al.* 2007). In random folding and balanced family folding, genomic relationships between training and validation samples are high while in family folding the relationships between training and validation samples are low as entire families were removed from the training set. The high breeding value accuracies observed in random and balanced family folding may be due to the genomic relationships captured by the markers while in family folding accuracies may be mainly due to the LD between markers and QTL as relationships between training and validation populations are low. Accuracy based on the LD between the markers and QTL would persist over many generations compared to the accuracy mainly due to capturing genetic relationships (Habier *et al.* 2010). However, both components of the accuracy are important to improve genetic gain through genomic selection (Habier *et al.* 2007).

In most genomic selection studies, accuracies are mainly due to genomic relationships captured by the markers resulting in overestimation of prediction accuracies (Beaulieu *et al.* 2014; Thavamanikumar *et al.* 2015; Gamal El-Dien *et al.* 2016, 2018; Thistlethwaite *et al.* 2017). There are very few studies estimating accuracies with genomic relationships removed between training and validation populations (Gamal El-Dien *et al.* 2016, 2018; Müller *et al.* 2017; Resende *et al.* 2017). Müller *et al.* (2017) observed reduction of accuracies close to zero in *Eucalyptus benthamii* and by more than half in *E. pellita* when the relationships between training and validation populations were minimized. Gamal El-Dien *et al.* (2016; 2018) also observed lower accuracies with family folding compared to random folding for GBLUP_A and GBLUP_AD models. In the present study, while the accuracies of the GBLUP_A model were lower with the family folding; they were however similar between the three methods for DBH and Vol with GBLUP_AD model (Table 5). The significant dominance ratios observed for these two traits (Tables 2 and 4) may explain the consistency of the accuracies between the three methods. Predictive abilities for DBH and Vol observed in this study with the GBLUP_AD model are similar to the accuracies of the mean annual increment (MAI) observed by Resende *et al.* (2017) with family folding cross-validation. While the predictive abilities between GBLUP_A and GBLUP_AD are found to be similar in Resende *et al.*’s (2017) study, they are however very low for the GBLUP_A model compared to the GBLUP_AD model in our study. This indicates the importance of dominance in estimating predictive ability in our study.

### Within-family selection

One of the main benefits of genomic selection in forest tree breeding is selecting superior trees in large full-sib families (Beaulieu *et al.* 2014). In tree breeding, mid-parental values are used for selecting top-ranking families in the absence of performance data from progeny trials. While better-performing families can be selected based on mid-parental values, these methods cannot be used for selecting superior genotypes within the selected families unless they are progeny tested. Genomic selection is ideally suited for making within family selections in the absence of performance data as markers can be used to capture the Mendelian segregation term. High within-family accuracies observed with family folding in this study indicate that it may be possible to select superior individuals in untested families derived from parents with a similar background to the training population. While predicting genetic values is more important in CP families than OP families, the selected trees with high genetic values may be used for establishing clonal trials from OP breeding programs.

### Predictions using markers from candidate genes

Accuracies of within-family predictions are mainly influenced by the marker-trait associations captured by the markers as differences in genomic relationships between the individuals within a family are small. For forward selections among sibs, markers that capture LD or marker-trait associations are essential (Thistlethwaite *et al.* 2019). Genomic predictions with informative SNPs from candidate genes may be important to capture the LD between markers and QTL. Genomic predictions using markers from exome capture revealed high accuracies but the accuracies are mainly due to genomic relationships captured by the markers rather than the marker-trait associations (Thistlethwaite *et al.* 2017, 2019). Similarly, the lower accuracies with family folding observed in Gama El-Dien *et al.*’s (2018) study compared to those in their 2016 study (Gamal El-Dien *et al.* 2016) may be due to random SNPs from the genotyping by sequencing (GBS) method used in their 2018 study. In the earlier study (Gamal El-Dien *et al.* 2016) they used SNPs within the genes. In the present study though, similar to Gama El-Dien *et al.* (2016) we have used pre-selected markers from candidate genes which may explain the relatively high accuracies in family folding compared to the results of Gamal El-Dien *et al.* (2018). In a previous study, we used preselected markers from disease genes to predict disease resistance to Teratosphaeria leaf disease (TLD) in *Eucalyptus globulus* (Thumma *et al.* 2017). High predictive ability (0.62) and high prediction accuracies (0.82) were observed using unrelated individuals in model training to predict disease resistance in another population at a different site. Similarly, Resende *et al.* (2017) used SNP markers from gene regions to predict within-family individuals in *Eucalyptus* hybrids. These results indicate the importance of using SNP markers from candidate genes to capture the short-range LD between markers and QTL which is an important component of genomic selection.

## Conclusions

In this study, we used ssGBLUP and GBLUP analyses to identify significant dominance effects underlying the growth traits in an OP population of *E. pellita*. High theoretical breeding value accuracies were observed for the three growth traits which are reflected in the high accuracies observed in cross-validation tests using all trees. As the number of trees genotyped is a small fraction of the total number of trees in this study, results from cross-validation were similar between ABLUP and ssGBLUP models with all the trees. Cross-validation tests within the genotyped trees also revealed high accuracies. Inclusion of dominance in prediction models improved the predictive abilities and prediction accuracies of DBH and Vol which exhibited high dominance ratios. High within-family accuracies with family folding indicate that the markers may be capturing the short-range LD between the markers and QTL as the relationships between training and validation populations are low. Markers from candidate genes may be important to capture the short-range LD. This study demonstrates the potential of genomic studies to unravel non-additive effects underlying complex traits in OP families which is not possible with the traditional methods.
